# Optimization of RAASi Therapy with New Potassium Binders for Patients with Heart Failure and Hyperkalemia: Rapid Review and Meta-Analysis

**DOI:** 10.3390/jcm10235483

**Published:** 2021-11-23

**Authors:** Andrea Montagnani, Stefania Frasson, Gualberto Gussoni, Dario Manfellotto

**Affiliations:** 1 Internal Medicine, Hospital of Grosseto, 58017 Grosseto, Italy; 2 FADOI Research Centre, 20123 Milan, Italy; stefania.frasson@fadoi.org (S.F.); gualberto.gussoni@gmail.com (G.G.); 3 Department of Internal Medicine, Hospital “Fatebenefratelli-AFaR”, 00186 Rome, Italy; dario.manfellotto@afar.it

**Keywords:** hyperkalemia, Patiromer, sodium zirconium cyclosilicate, meta-analysis, heart failure, RAAS

## Abstract

(1) Background: The objective of this rapid review is to assess whether new potassium binders (NPBs) could enable the optimization of RAASi therapy more than usual care or placebo in patients with or at risk of heart failure and hyperkalemia. (2) Methods: We searched for RCTs that included patients with or at risk of hyperkalemia and patients treated with Patiromer or sodium zirconium cyclosilicate (ZSC). The comparators were placebo, usual care, and potassium binders with different doses or different treatment protocols. We searched the Cochrane CENTRAL, MEDLINE, and ClinicalTrials.gov databases. The risk of bias was assessed using the Cochrane risk of bias tool for RCTs. Data were pooled using the random effects model, and the fixed effects model was used for sensitivity analysis. (3) Results: We included 12 studies with 2800 enrolled patients. Only three of these trials (412 patients) were included in the meta-analysis. NPBs seemed to have an effect on the optimization of MRA therapy, with an RR (95% CI) of 1.24 (1.09, 1.42) (moderate certainty evidence); Patiromer seemed to have an effect on MRA optimization, with an RR (95% CI) or 1.25 (1.08, 1.45) (high certainty evidence). ZSC seemed to have no effect on enabling MRA therapy, with an RR (95% CI) of 1.19 (0.89, 1.59) (low certainty evidence). The AEs in HF patients with hyperkalemia treated with Patiromer were GI disorders and hypomagnesemia. ZSC The AEs included chronic cardiac failure, hypokalemia, and edema. (4) Conclusions: This meta-analysis included three studies with a small number of patients and a short follow-up period (1–3 months). The evidence of the effect of NPBs on MRA optimization had a moderate certainty for imprecision. Data on the effect on MRA optimization and less severe AEs in long-term treatment seem to suggest the use of Patiromer for the optimization of MRA therapy in patients with or at risk of heart failure and hyperkalemia. Future adequately powered RCTs are needed to assess the benefits and potential harms of potassium binders.

## 1. Introduction

### 1.1. Description of the Condition

RAASi therapy (ACEi, ARB, MRA, and ARNi therapy) is the cornerstone therapy for patients with heart failure with reduced ejection fraction (HFrEF). International guidelines recommend RAAS inhibitors because of strong evidence [1,2]. Even so, there are some barriers to the implementation of this therapy, including hypotension, kidney dysfunction, and hyperkalemia (HK) [3]. HK is defined as high serum potassium (K^+^) levels. There is no universal cutoff for HK, but a level of 5.0 mEq/L is common [4]. HK is a life-threatening condition that leads to significant morbidity and mortality. A recent evaluation of medical records demonstrated an increase in all-cause mortality both with K^+^ values below the normal range and values higher than 5.5–6.0 mEq/L, with a clear effect of comorbidities, such as diabetes, heart failure, and CKD, considered individually or together [5]. In three pivotal clinical studies examining the efficacy of RAAS inhibitors in heart failure, HK occurred in these patients at a non-negligible frequency, although patients with a high risk of HK (i.e., with impaired renal function) were excluded from the studies [6,7,8]. 

The latest ESC guidelines recommend that RAAS inhibitors should be administered at the maximum recommended dose in order to achieve a positive effect on mortality in patients with HFrEF [1]. The protective effect of RAAS inhibitors on mortality in patients with heart failure is maximized when used in combination, but at the same time, a higher risk of HK, and therefore a worse outcome, is caused by using a combination with a sub-optimal dose of RAASi. In addition, patients who appear to benefit from the administration of RAAS inhibitors also have a higher risk of HK [9]. Recently, Rossignol et al. published data from 9222 patients with chronic heart failure in the ESC-HFA-EORP Heart Failure Long-Term Registry, confirming the association between HK and mortality. However, when the same data were adjusted for RAAS inhibitor discontinuation, HK was no longer associated with mortality, suggesting that HK may be a risk marker for RAAS inhibitor discontinuation rather than a risk factor for poorer outcomes [10]. In an unpublished survey that the Federation of Hospital Internists (FADOI) carried out among approximately 300 Italian internists in 2020, RAAS inhibitors were discontinued by 80% of physicians when potassium was more than 5.5 mEq/L or down-titrated when potassium serum values were higher than 5.0 mEq/L. This conservative and cautious approach seems to be common since HK is correlated with higher mortality; however, it induces a lack of optimization of therapy in patients with heart failure and/or CKD who are treated with RAAS inhibitors, with a consequent increase in mortality [11].

### 1.2. Description of the Intervention

Two new drugs have been developed to treat HK: Patiromer and sodium zirconium cyclosilicate (SZC). Patiromer is a spherical, non-resorbed, metal-free, cross-linked fluoroacrylate polymer [12]. Sodium zirconium cyclosilicate is a non-absorbed, non-polymeric inorganic powder with a uniform microporous structure [13].

### 1.3. How the Intervention Might Work

Most potassium is eliminated through the kidneys, but 5–10% is eliminated in the colon. Both Patiromer and SZC remove potassium by exchanging cations (calcium and sodium for Patiromer and SZC, respectively) for potassium in the gastrointestinal tract, binding potassium and increasing its fecal excretion. Patiromer was developed to provide a higher potassium binding capacity compared with the polystyrene sulfonate polymer. It has improved physical properties, such as a low swell ratio that allows for minimal water uptake, and, importantly, Patiromer uses calcium rather than sodium as the exchange cation. Patiromer was completely ionized for optimal ion exchange at the physiological pH of the large intestine, where the potassium concentration in the gastrointestinal tract was the highest [12]. Sodium zirconium cyclosilicate is highly selective for potassium ions in vitro, even in the presence of other cations, such as calcium and magnesium. Sodium zirconium cyclosilicate traps potassium throughout the gastrointestinal tract (GI) and reduces the concentration of free potassium in the GI lumen, thereby lowering serum potassium and increasing fecal potassium excretion to resolve HK [13].

### 1.4. Why It Is Important to Do This Review

Although several systematic reviews have examined the efficacy of these new compounds in lowering serum potassium [14,15,16,17], no SRs have analyzed whether NPBs allow the optimization of RAASi therapy in HF patients. This is the first quick review in this area.

### 1.5. Objectives 

The objective of this rapid review was to assess whether new potassium binders (NPBs) could enable the optimization of RAASi therapy more than usual care or placebo in patients with or at risk of heart failure and HK.

## 2. Materials and Methods

### 2.1. Criteria for Considering Studies for this Review 

#### 2.1.1. Types of Studies

We included RCTs published in English before the end of July 2021; we also included studies that were not published but had results available in the international registry of clinical trials. We chose only RCTs because this was a reference design study for the evaluation of treatments.

#### 2.1.2. Types of Participants

We included studies with patients with or at risk of HK.

#### 2.1.3. Types of Interventions 

We included patients treated with a Patiromer or sodium zirconium cyclosilicate. The comparators in the studies were placebo, usual care, and potassium binders with different doses or different treatment protocols. We excluded studies with SPS and CPS and extension studies without comparators.

#### 2.1.4. Types of Outcome Measures 

Primary outcomes:

- Proportion of patients on MRA therapy (guideline target dose) compared with placebo at the end of the study
- Proportion of patients on ACE/ARB therapy (guideline target dose) compared with placebo at the end of the study

Secondary outcomes

- Proportion of patients on ARNi therapy (guideline target dose) compared with placebo at the end of the study
- Safety of treatments in patients with heart failure

### 2.2. Search Methods for Identification of Studies 

Electronic searches: We searched data in Cochrane CENTRAL, MEDLINE (via PubMed), and ClinicalTrials.gov. We searched for the terms “Patiromer” or “sodium zirconium cyclosilicate”. In PubMed, we used (“Patiromer” [Supplementary Concept]) OR “sodium zirconium cyclosilicate” [Supplementary Concept]. In the Cochrane Library, we searched the terms “Patiromer in Title Abstract Keyword OR sodium zirconium cyclosilicate in Title Abstract Keyword” (word variations were searched).

### 2.3. Data Collection and Analysis 

#### Selection of Studies

We used a standardized title and abstract form of title and abstract screening; two reviewers conducted dual screening of at least 20% of the abstracts with conflict resolution, and one reviewer conducted the screening of the remaining abstracts. A second reviewer checked all excluded abstracts and resolved the conflicts. One reviewer checked all included full-text articles, and a second reviewer checked all excluded full-text articles.

### 2.4. Data Extraction and Management 

We recorded the number of HF patients in the studies, treatments (type and dose), comparators, and patients on RAASi at the end of the study. Data were extracted from the original publication or the substudy publication. 

### 2.5. Assessment of Risk of Bias in Included Studies

The risk of bias was assessed using the Cochrane Risk of Bias tool for RCTs [18]. A single reviewer rated the risk of bias, with a full verification of all judgments (and support statements) conducted by a second reviewer. We limited the risk of bias ratings to outcomes.

### 2.6. Measures of Treatment Effect 

For dichotomous outcomes (optimization of MRA, ACEi/ARB, and ARNi therapies), the results are expressed as risk ratios (RRs) between groups with 95% confidence intervals (CIs).

### 2.7. Assessment of Heterogeneity 

We assessed clinical heterogeneity by determining whether participant characteristics, interventions, outcomes measurements, and timing of outcome measurements were similar across the studies. We assessed the heterogeneity by visual inspection of the forest plots. Statistical heterogeneity was quantified using the I2 statistic, which describes the percentage of total variation between studies due to heterogeneity and not due to sampling errors [19]. We interpreted the I2 statistic as an approximate guideline as follows: 0% to 40%, no important heterogeneity; 30% to 60%, moderate heterogeneity; 50% to 90%, considerable heterogeneity; and 75% to 100%, considerable heterogeneity.

The significance of the observed value of I2 depends on the size and direction of the treatment effects and the strength of the evidence for heterogeneity (e.g., a *p*-value for the Chi-square test or a confidence interval for I2) [18]. If there was considerable heterogeneity, we examined the data further by comparing the characteristics of individual studies and subgroup analyses.

### 2.8. Assessment of Reporting Biases 

To assess the publication bias, we created funnel plots when at least 10 studies examining the same treatment comparison were included in the review and commented on whether an asymmetry in the funnel plot was a publication bias or a methodological or clinical one. In order to evaluate potential effects in small studies in the meta-analysis (i.e., the intervention effect was more beneficial in smaller studies), we compared the effect estimates derived from a random effects model and a fixed effects model of the meta-analysis. In the case of small study effects, the random effects model could provide a more favorable estimate of the intervention than the fixed effects estimate.

### 2.9. Data Synthesis 

The data were pooled with the random effects model, but the fixed effects model was used to ensure the robustness of the chosen model and the susceptibility to outliers. We used Review Manager (RevMan) (computer program) version 5.4, Cochrane Collaboration 2020 (Cochrane, London, UK).

### 2.10. Subgroup Analysis and Investigation of Heterogeneity 

A subgroup analysis was performed to examine the possible sources of heterogeneity (if sufficient data were available). We chose to use the types of potassium binders as subgroups.

### 2.11. Sensitivity Analysis 

We carried out sensitivity analyses in order to examine the influence of the effects model (random vs. fixed) on the effect size. 

We also carried out sensitivity analyses to examine the influence of the following factors on the effect size:

- Repeating the analysis with the fixed effects model
- Repeating the analysis excluding studies with high risks of bias

## 3. Results

### 3.1. Description of Studies 

The electronic search results identified 334 records. After duplicating elimination, 159 records remained. We excluded 29 records: 25 were ongoing, 2 were unknown, and 2 had no results. After title and abstract screening of 130 records, we identified 12 studies (24 records). We excluded 106 full-text articles for the following reasons: four clinical cases, 11 comments, seven editorials, 10 letters, five systematic reviews, two observational studies, 51 reviews, five different languages, and five studies on animals.

**Figure 1:**
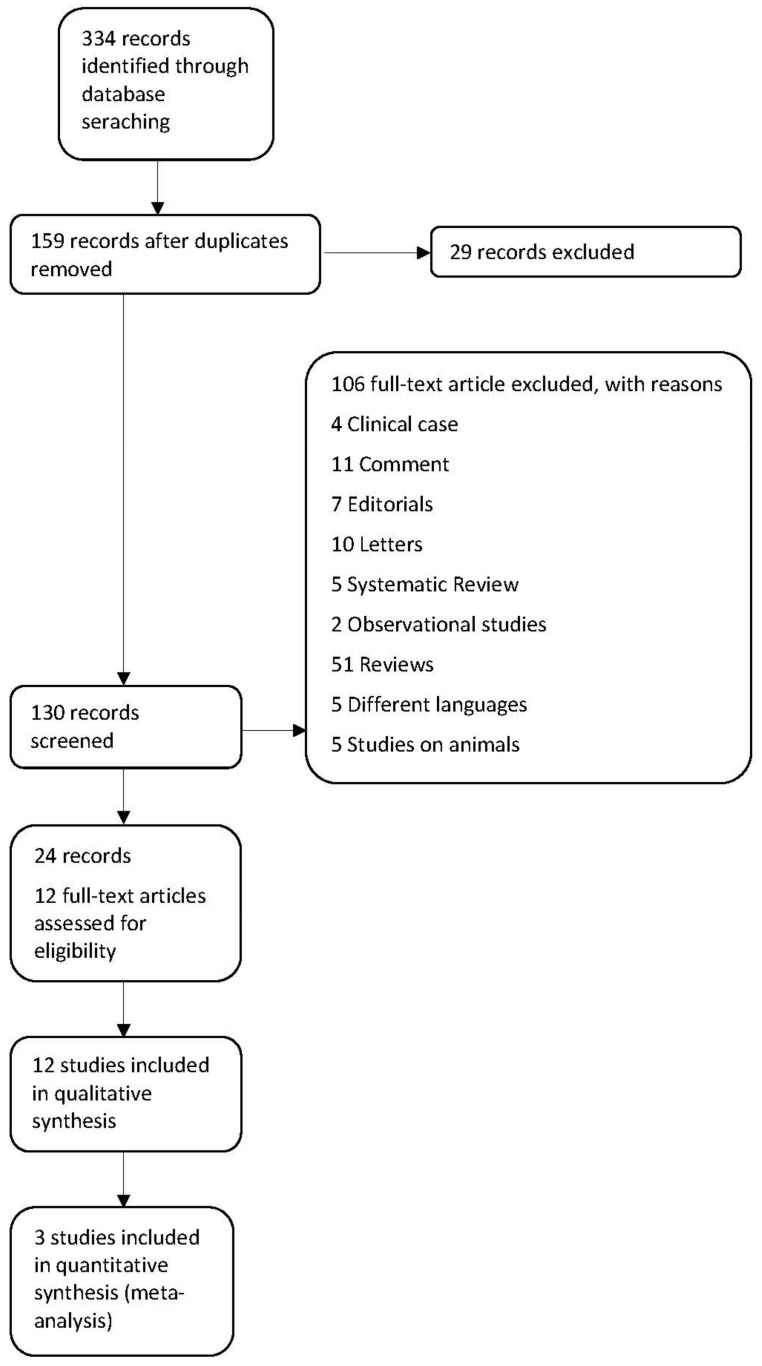
PRISMA flowchart.

We included 12 studies with 2800 patients enrolled. Only four studies included data on the use of RAAS inhibitor therapy at the end of the study (PRIORITIZE HF, AMBER, PEARL HF, and OPAL HK). Data on MRA therapy was present in 3 studies (PRIORITIZE HF, AMBER, and PEARL HF, 412 patients) and we included them in the meta-analysis. Data on the use of ACEi/ARB therapy in OPAL HK was reported separately. For two studies with Patiromer (AMBER and OPAL HK), we extracted data from publications on subgroups with heart failure [20,21] (Figure 1).

### 3.2. Included Studies 

The studies included in the review are reported in Table 1.

### 3.3. Excluded Studies 

We did not exclude any studies.

### 3.4. Risk of Bias in Included Studies the Review

**Figure 2:**
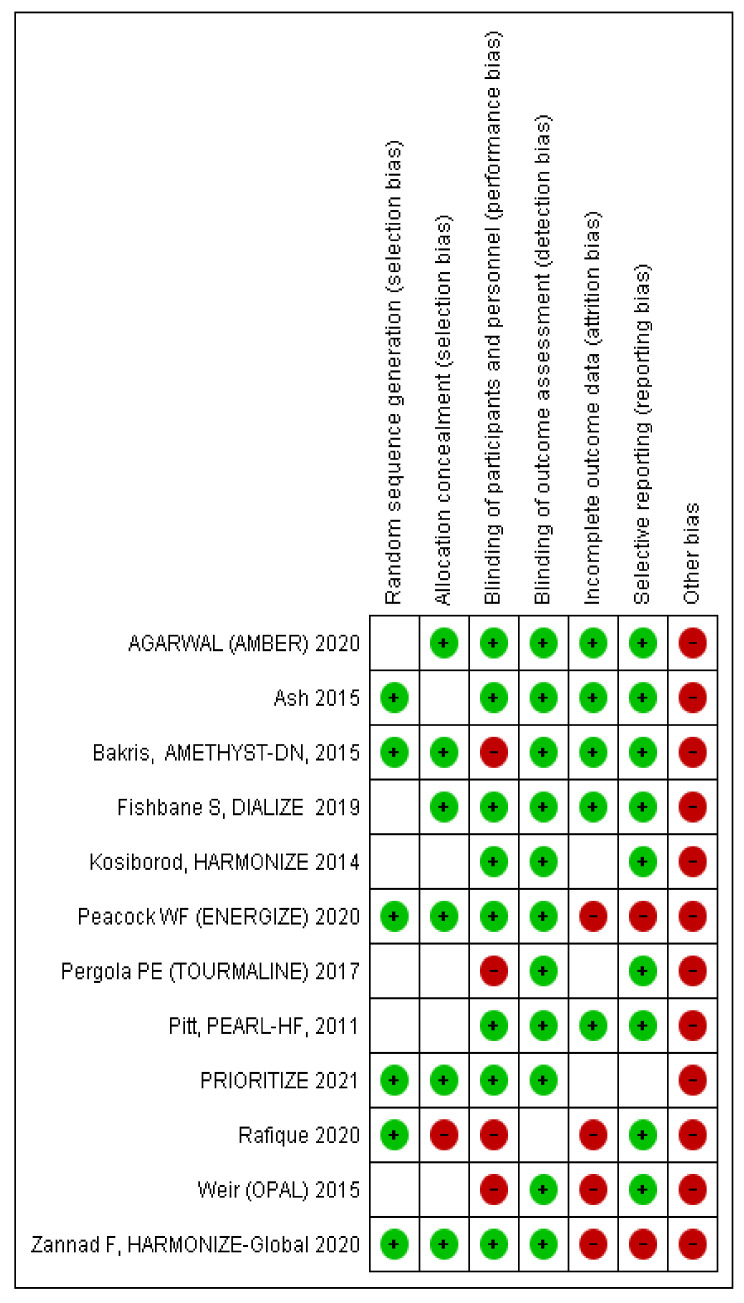
Risk of bias.

The risk of bias is reported in Figure 2.

### 3.5. Selective Reporting (Reporting Bias) 

We could not make a funnel plot because we had only three studies on the principal outcome.

### 3.6. Other Potential Sources of Bias 

All the studies were funded by sponsors, and this may be a source of bias.

### 3.7. Effects of Interventions 

**Figure 3:**
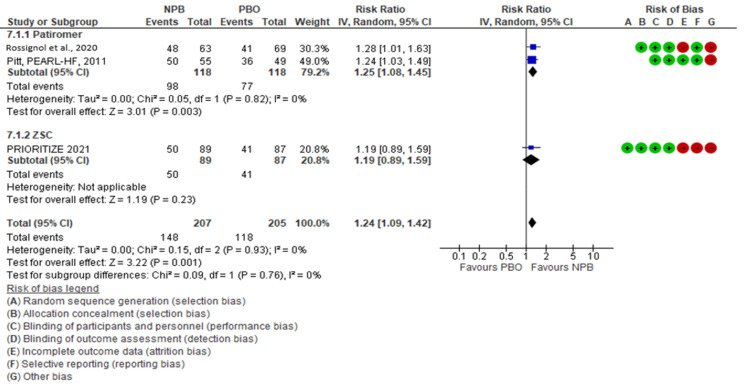
MRA optimization.

NPBs seemed to have an effect on the optimization of MRA therapy, with a risk ratio (95% CI) of 1.24 (1.09, 1.42); there was no heterogeneity (I^2^ = 0%, moderate certainty evidence). Patiromer seemed to have an effect on MRA optimization; in two studies with 232 patients, the risk ratio (95% CI) was 1.25 (1.08, 1.45) with I^2^ = 0 (high certainty evidence). ZSC seemed to have no effect on MRA optimization, as shown by one study with 176 patients (risk ratio (95% CI)), 1.19 (0.89, 1.59) (low certainty evidence)) (Figure 3). The subgroup analysis did not show heterogeneity between the subgroups (I^2^ = 0%). 

**Figure 4:**
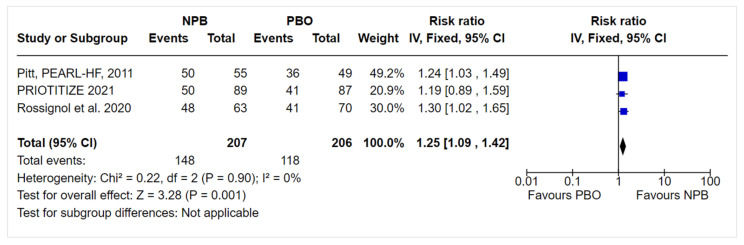
Sensitivity analysis.

We conducted a sensitivity analysis of the MRA optimization outcome. The fixed effects model did not change the magnitude or direction of effect: the NPB RR (95% CI) was 1.25 (1.09, 1.42) (Figure 4); the same was observed for the exclusion of the study with a high risk of bias, with an RR (95% CI) of 1.25 (1.08, 1.45).

Optimization of ACEi/ARB therapy: There was only one study with Patiromer; it did not seem to have an effect on ACEi/ARB optimization; in 49 patients, the risk ratio (95% CI) was 1.20 (0.85, 1.67) (low certainty evidence).

Optimization of ARNi therapy: No data were available.

### 3.8. Safety 

Patiromer: The most common adverse events were gastrointestinal disorders (12/56 PEARL HF, 9/105 AMETHYST-DN, 5/27 OPAL and 7/63, Rossignol et al., 2020), and hypomagnesemia (13/56 PEARL HF, 19/105 AMETHYST-DN, 3/102 OPAL, and 2/63 Rossignol et al., 2020) [21]. 

ZSC: The most common adverse events were mild hypokalemia (S-K < 3.5 mmol/L, 4/61 HARMONIZE, 7/91 PRIORITIZE), edema (8/61 HARMONIZE, 1/90 PRIORITIZE), and chronic cardiac failure (MEDRA definition) (9/91 PRIORITIZE).

## 4. Discussion

We identified 12 studies randomizing 2800 adult participants evaluating potassium binders for chronic HK (defined as >5 mmol/L). Data for efficacy outcomes included in this review were available in four of these twelve studies: three for MRA therapy (PRIORITIZE HF [30], PEARL HF [29], and Rossignol et al., 2020 [21]) and only one for ACEi/ARB therapy (OPAL HK [32]). In the safety analysis, we included six studies (PRIORITIZE HF [30], HARMONIZE [34], PEARL HF [29], Rossignol et al., 2020 [21], OPAL HK [32], and AMETHYST-DN [24]). New potassium binders seem to have an effect on MRA optimization (moderate certainty evidence). Patiromer seems to have an effect on MRA optimization, while ZSC seems to have no statistically significant effect. It should be noted that the PRIORITIZE study was prematurely terminated because of the COVID-19 pandemic, resulting in a reduced sample size and a high premature treatment discontinuation rate. A sensitivity analysis showed that the evidence was robust; the fixed effects model and random effects model did not change the result. The same happened with the exclusion of the PRIORITIZE study because of the high risk of bias.

The effect of Patiromer on ACEi/ARB optimization was not statistically significant, but the direction of the effect was in accordance with the results of the MRA outcome. MRA therapy is the most discontinued therapy for HK, and the efficacy of its optimization is the answer to unmet medical needs.

The AEs in HF patients with HK treated with Patiromer were similar to those in hyperkalemic patients: GI disorders and hypomagnesemia. The latter had a different incidence between studies in which different doses were used. For ZSC, the most common AEs in hyperkalemic patients included in the RCT were hypokalemia and edema. In our review, hypokalemia was present with an incidence similar to that in other studies in patients without heart failure, while edema was lower. However, chronic cardiac failure (MEDRA definition) was present. The presence of sodium counter-ions in ZSC (400 mg in 5 g of product) and not in Patiromer may have caused this difference in HF patients. More studies are needed to better understand the long-term safety of ZSCs [35].

### 4.1. Overall Completeness and Applicability of Evidence

There are only four studies with outcomes on RAASi optimization, and all had a limited time of follow-up (1–3 months) with a small number of patients included (99–191). Evidence of the efficacy of NPBs for the initiation and up-titration of MRA therapy has extended the ability of these new substances to maintain RAASi therapy in patients with heart failure. The evidence for the effect of Patiromer on outcomes had high certainty and is in accordance with the effect in patients with HK without HF. The evidence for ZSC had low certainty, and future studies, such as the LIFT study [36], may change the results.

### 4.2. Quality of the Evidence

We used the GRADE method to assess the quality of the results [37]. The evidence of the effect of NPB on MRA optimization had moderate certainty for imprecision. The effect of Patiromer on MRA optimization had high certainty. The effect of ZSC on outcomes had low certainty for imprecision and risk of bias. The effect of Patiromer on ACEi/ARB therapy had very low certainty for imprecision and risk of bias.

### 4.3. Potential Biases in the Review Process

The authors adopted the following definition of an RR: ‘‘A rapid review is a form of knowledge synthesis that accelerates the process of conducting a traditional systematic review through streamlining or omitting various methods to produce evidence for stakeholders in a resource-efficient manner.’’ The authors followed the recommendations reported in Garrity (2021). All the studies included in the RR were supported by sponsors and were impossible to exclude from the RR. The limited number of studies was a constraint on our ability to assess potential reporting bias and selective outcome reporting. The effects of potassium binder interventions on longer-term outcomes were uncertain, and the treatment endpoint was the use of RAASi therapy; if this strategy improves the clinical outcome, a well-designed RCT is needed.

### 4.4. Agreements and Disagreements with Other Studies or Reviews

The reviews published to date have studied the effect of NPBs on serum potassium levels. The focus of our review was to enable RAASi therapy in patients with HF and HK, and it is the first review in this area.

## 5. Conclusions

### 5.1. Implications for Practice

The results of our review indicated that NPBs seem to have an effect on MRA optimization with moderate certainty of evidence. Patiromer seems to have an effect on MRA optimization (high certainty of the evidence), while ZSC seems to have no effect on optimizing MRA therapy (low certainty of evidence).

The effect, quality of evidence, and less damaging AEs in long-term use seem to suggest the use of Patiromer for the optimization of MRA therapy in patients with or at risk of heart failure and HK. This different use of NPB is the same as that reported in other systematic reviews of patients with chronic or recurrent HK [14,15]. In the future, this suggestion could change if the publication of new data on the efficacy and safety of ZSC changes its risk/benefit profile.

### 5.2. Implications for Research

There is a gap in knowledge about optimizing ARNi therapy in patients with heart failure and HK and in a head-to-head study between NPBs. Future adequately powered RCTs are needed to assess the benefits and potential harms of potassium binders, and if their use will lead to the optimized use of RAAS inhibitors to improve clinical outcomes in patients with heart failure and HK.

## Figures and Tables

**Table 1:** Studies included in the review.

Study ID	Population	Intervention	Comparator	Outcome
Agarwal, (AMBER) 2020 [22].	295 patients with CKD (eGFR 25 to 45 mL/min/1.73 m^2^) and resistant hypertension, baseline K^+^ levels (4.3 to 5.1 mmol/L)	Patiromer (8.4 g o.d.)	Placebo	Proportion of patients remaining on spironolactone
Ash, 2015 [23].	90 outpatients, CKD stage 3 and hyperkalemia (5.0 to 6.0 mEq/L)	0.3 g, 3 g, 10 g of ZSC	Placebo	Rate of serum K^+^ decline in the first 48 h
Bakis, AMETHYST-DN, 2015 [24]	306 patients with T2DM (eGFR, 15 to <60 mL/min/1.73 m^2^ and serum K^+^ level >5.0 mEq/L).	Mild hyperkalemia: 4.2 g, twice daily; moderate hyperkalemia: 8.4 g, twice daily	Mild hyperkalemia: 8.4 g, or 12.6 g twice daily; moderate hyperkalemia: 12.6 g, or 16.8 g twice daily	Mean change in serum K^+^ from baseline to week 4 and adverse events through 52 weeks
Fishbane, DIALIZE 2019 [25].	97 patients with ESRD and HD three times weekly, predialysis sK^+^ > 5.4 mmol/L after the long interdialytic interval as well as predialysis sK^+^ > 5.0 mmol/L after at least one short interdialytic interval	ZSC 5 g, 10 g, 15 g	Placebo	Patients with predialysis serum K^+^ of 4.0–5.0 mmol/L
Kosiborod, HARMONIZE 2014 [26].	258 patients with hyperkalemia (serum potassium ≥ 5.1 mEq/L)	ZSC 10 g three times a day for 48 h (correction phase), after which normokalemic patients (3.5–5.0 mEq/L) were randomized to ZSC, 5 g, 10 g, or 15 g daily (Maintenance phase)	Placebo	Mean serum potassium level in each zirconium cyclosilicate group vs. placebo in MP
Peacock WF, ENERGIZE 2020 [27].	70 ED patients with blood potassium ≥ 5.8 mmol/L	SZC 10 g	Placebo	Mean change in level of serum K^+^ from baseline until 4 h after the starting dose
Pergola, TOURMALINE, 2017 [28].	112 patients with serum K^+^ ≥ 5.0 mEq/L	Patiromer (by 8.4 g/day–25.2 g/day) with food	Patiromer (8.4 g/day–25.2 g/day) without food	Proportion of patients with level of serum K^+^ in the range of 3.8–5.0 mEq/L at week 3 or 4
Pitt, PEARL-HF, 2011 [29].	105 HF patients with CKD (eGFR <60 mL/min) or a history of HK	Patiromer 25.2 g	Placebo	Change from baseline in level of serum K^+^ at the end of study
PRIORITIZE HF 2021 [30]	182 HF patients with serum potassium > 5.0 mmol/L or at high risk of hyperkalemia	ZSC 5 g or 10 g	Placebo	Percentage of patients receiving ACEi, ARB, MRA, or ARNi treatments at month 3
Rafique 2020 [31].	30 ESRD patients, serum K^+^ ≥ 6.0 mEq/L	Patiromer 25.2 g + SOC	SOC	Difference in serum K^+^ between groups at 6 h
Weir, OPAL, 2015 [32]	Initial phase: 243 CKD patients in RAASi and with serum K^+^ levels between 5.1 and 6.5 mmol/L. Withdrawal phase: 105 patients in normokalemic range	Patiromer 4.2 g or 8.4 g twice/day	Placebo in withdrawal phase	Initial phase: mean change in the serum K^+^ level from baseline to week 4. Withdrawl phase: between-group difference in the median change in the serum K^+^ level
Zannad, HARMONIZE GLOBAL, 2020 [33].	267 patients with serum K^+^ ≥ 5.1 mmol/L	CP: ZSC 10 g 3 times a day for 48 h. MP: ZSC 5 g to 10 g	Placebo in MP	Mean serum K^+^ level during days 8–29 of the MP

The characteristics of the trials included in the meta-analysis are reported in Table 2.

**Table 2:** Studies included in the meta-analysis.

Study ID	Design of Study	Outcome	Duration	MRA NPB (End of Study)	MRA PBO (End of Study)
Rossignol et al., 2020 [21]	RCT	Patients taking spironolactone at week 12	3 months	48/63	41/69
PEARL HF, Pitt B et al., 2011 [29]	RCT	Change in serum K^+^ from baseline to day 28	28 days	50/55	36/49
PRIORITIZE-HF, 2021 [30]	RCT	Percentage of patients receiving different categories of RAASi	3 months	50/89	41/87

## Data Availability

Not applicable.
